# Suicide related to the COVID-19 pandemic in India: A systematic review

**DOI:** 10.1007/s44202-022-00063-1

**Published:** 2023-01-10

**Authors:** Firoj Al-Mamun, Mark Mohan Kaggwa, Ismail Hosen, Md. Tajuddin Sikder, Mark D. Griffiths, Mohammed A. Mamun

**Affiliations:** 1 CHINTA Research Bangladesh, Dhaka, 1342 Savar, Bangladesh; 2 Department of Public Health and Informatics, Jahangirnagar University, Dhaka, Savar, 1342 Bangladesh; 3 Department of Public Health, University of South Asia, Dhaka, Savar, Bangladesh; 4 Department of Psychiatry, Faculty of Medicine, Mbarara University of Science and Technology, Mbarara, Uganda; 5 International Gaming Research Unit, Psychology Department, Nottingham Trent University, Nottingham, UK; 6 Department of Psychiatry and Behavioural Neurosciences, McMaster University, Hamilton, Ontario Canada

**Keywords:** COVID-19 suicide, Suicide, Suicidal behavior, Suicide risk factors, Suicide methods, Media reported suicide

## Abstract

**Background:**

The suicide rate has increased during the pandemic in India. Moreover, several studies, especially press-media reporting suicide studies have been conducted but no systematic review has been attempted in this context. Therefore, the present study systematically investigated the risk factors associated with suicidal behaviors, and the method of suicide during the COVID-19 pandemic in India.

**Methods:**

Following the PRISMA guidelines, a systematic search was performed to include papers published up until September 30, 2022. From an initial 144 papers, 18 studies which met the inclusion criteria were included in the present review. The Pierson’s method was used for quality assessment of the included studies in the present review.

**Results:**

The risk factors associated with suicide comprised: (i) socio-demographic factors (e.g., being aged between 31 and 50 years, male, married, unemployed), (ii) behavior and health-related factors (e.g., unavailability of alcohol and alcohol withdrawal symptoms, poor state of physical health and health issues, family disputes, relationship complexities, and sexual harassment), (iii) COVID-19-related factors (e.g., fear of COVID-19, COVID-19 test results, quarantine or isolation, financial hardship due to the pandemic, having influenza-like symptoms, experiencing stigmatization and ostracism despite testing negative, separation from family due to transport restrictions, misinterpreting other illness symptoms as COVID-19, saving the village from infection, watching COVID-19 videos on social media, online schooling, perceived stigma toward COVID-19, and being suspected of having COVID-19), and (iv) psychopathological stressors (depression, loneliness, stress, *TikTok* addiction, and poor mental health, suicidal tendencies, helplessness, and worrying). Hanging was the most common method of suicide. In addition, jumping from high buildings, poisoning, drowning, burning, cutting or slitting throat or wrists, self-immolation, medication overdose, electrocution, pesticide, and gun-shot were also used to carry out the suicide.

**Conclusions:**

Findings from this research suggest multiple reasons for suicide during the COVID-19 pandemic and knowledge of such factors could aid in developing suicide prevention strategies focusing the most vulnerable cohorts inside and outside India.

## Introduction

Suicide is a major multifactorial phenomenon, which includes diverse factors that result in death by suicide. More than 20 suicide attempts are typically reported before a successful suicide attempt (i.e., suicide completion) [1]. Low and middle-income countries like India have been reported to have approximately 80% of the global suicide occurrences [2]. Additionally, the suicide rate in India has been increasing based on the National Crime Records Bureau (NCRB) data [3]. For instance, a report showed that more than 139,000 suicides in India were recorded in 2019 with a 3.4% increase compared to 2018 (134,516 suicides) which was higher than 2017 (129,123 suicides) [3]. In addition, statistics have shown that there were more than 380 daily suicide cases in India in 2019 [3]. Given the increasing numbers of suicides, the incidence rate may also rise during stressful situations such as the COVID-19 pandemic.

During the COVID-19 pandemic, individuals have reported to be suffering psychologically due to lockdown-related stressors such as fear and panic, frustration, scarcity of basic supplies, the authenticity of reliable information, perceived stigma, financial distress, and lack of physical exercise [4–9]. Such factors heighten the chances of suicide and suicidal behaviors [10]. In prior outbreaks, suicide has been reported to increase because of these stressors. Some of the studies claimed that the suicide rate increased during the COVID-19 pandemic due to unemployment. India has already reported severe economic disruption due to the pandemic [11]. Given the situation that the country has already experienced, the second wave of the pandemic intensified the problems. For instance, an Indian study claimed suicide had increased during the pandemic based on press media reports [12]. However, evidence utilizing data from other countries’ national suicide databases did not find an increase in suicide rate in 21 countries [13].

On 12 February 2020, Goyal et al. [14] reported the first Indian suicide case related to the COVID-19 pandemic. A 50-year-old man died by hanging on a tree, where the fear of COVID-19 infection was alleged to be the suicide stressor. The victim was reported being constantly obsessed with the videos of the Chinese suspected patients’ forcefully being placed into healthcare settings for quarantine against their will. He reported experiencing flu-like symptoms to a physician, which resulted in disturbed thinking concerning the protection of his family. He ended up quarantining himself, and the fear and panic of acquiring COVID-19 appeared to have been the main reasons to end his life [14]. One month after this case was reported, another study investigated a total of 69 Indian suicide cases between March to May 2020 and reported that fear of COVID-19 appeared to be the most significant suicide attributor [15]. Given the unprecedented situation, a number of studies have continued to investigate COVID-19 related suicide in India. However, to the best of the present authors’ knowledge, there has been no integration of the findings of these studies. Therefore, the present review explored the stressors that lead to suicide, and the methods used to commit suicide among Indian individuals during the COVID-19 pandemic.

## Methods

### Search strategy

To conduct the present a systematic review, the Preferred Reporting Items for Systematic Reviews and Meta-Analyses (PRISMA) guidelines were followed [16]. First, a systematic literature search was performed using *PubMed, Scopus,* and *EuroPMC* to retrieve Indian suicide studies related to the COVID-19 pandemic. Additional searches were conducted using *Google Scholar,* and *ResearchGate* to collect the preprints. Search keywords included: press-media reporting suicide, press-media suicide, media suicide, press-report suicide, COVID-19 suicide India, pandemic suicide India. These were combined following the Boolean operator (OR, AND, NOT).

### *Study selection criteria*

First, the titles and abstracts of each paper were examined. Then the available full-text papers were assessed based on the inclusion criteria. The inclusion criteria were (i) being a study conducted in India, (ii) reporting a single case or case series, (iii) being published since the start of the pandemic to September 30, 2022, (iv) being either related to suicide case(s) or suicide behaviors (i.e., suicidal ideation, suicide plan, and suicide attempt), (v) reporting stressors for planning, attempting or completing suicide, (vi) being published in a peer-reviewed journal or preprint, and (vii) being published in the English language.

### *Data eligibility*

**Fig. 1:**
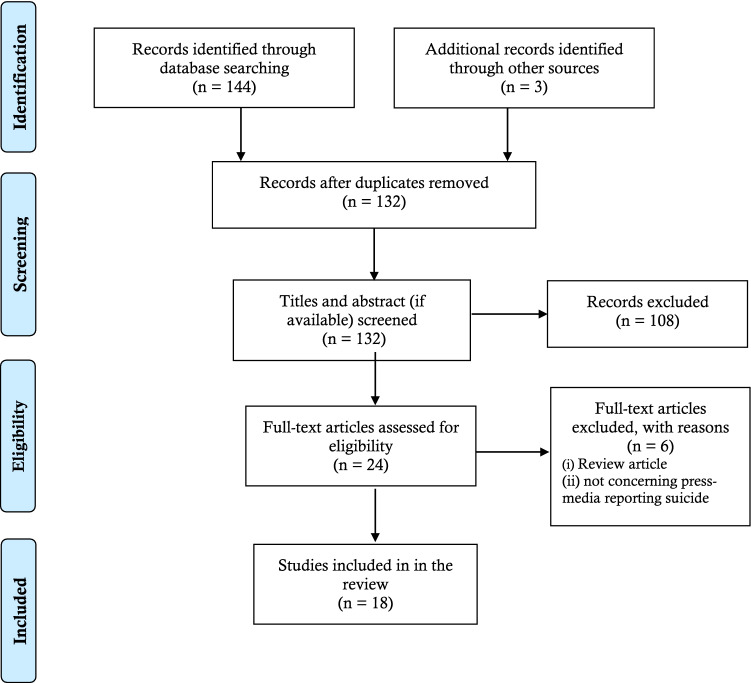
PRISMA flow-chart diagram

From several databases, 144 papers were collected, with 132 papers remaining after removing duplicates. After eliminating 111 papers based on the content of the titles and abstracts, 24 papers remained for full reading. After assessing the eligibility of the full-text papers, 18 papers were included in the final review. Six papers were excluded because they were either (i) a review paper (n = 1) or (ii) did not report suicide cases (n = 5) (Fig. 1).

### *Data extraction*

A data extraction file was generated to collate information from the papers. Data extraction included: (i) first author and publication year, (ii) sampling procedure, (iii) data collection reporting time, (iv) the number of cases, (v) gender, (vi) age (years), (vii) suicide stressors, and (viii) methods of suicide. The first pair of authors independently screened the title and abstract, reviewed the full text, included relevant papers, and extracted data from the full text. Title and abstract screening and removing duplicate papers were completed in *Mendeley* software. If any discrepancies occurred this was resolved by consulting with all the authors.

### *Quality assessment*

The quality assessment of the included studies was carried out using the Pierson’s method. The method has five components comprising documentation, uniqueness, educational value, objectivity, and interpretation which were used to assess the quality and validity of case reports/case series studies. Each component has a maximum of two points (score range from 0 to 2). Therefore, the total score ranged from 0 to 10, where 9–10 indicated high quality, 6–8 indicated moderate quality, and ≤ 5 indicated low quality [17].

## Results

### *Description of the included studies*

A total of 18 press-reporting studies were included in the present review based on the inclusion criteria [6–8, 12, 14, 15, 18–30]. All of the studies were ‘media reporting suicide’ studies, where data of the suicide victims were retrospectively collected. Most of the studies used *Google Search* to collect suicide victim data during the pandemic period between January 2020 and September 2020. A total of three case studies and 15 case series studies were conducted on different cohorts such as the general public, adolescents and youth, healthcare professionals, and celebrities, while the remaining studies did not report any cohort. Only one study [26] reported the victims’ mean age. The age of the victims in the other studies ranged from 11 to 80 years. All of the studies reported reasons for suicide or stressors, with all but three studies reporting the method of suicide (Table 1). Most of the studies had a low quality assessment score **(**Table 2**)**.

**Table 1:** Characteristics of the included studies

First author(s) and publication year	Sampling procedure; Population	Data collection reporting time	Case (s)	Gender	Age (years)	Suicide Stressors	Methods of suicide (n/%)
Dsouza et al. (2020)	Purposive; NR	March to May 24, 2020	69	Male (n = 63) and Female (n = 6)	19 to 65 years	Fear of COVID-19 infection (n = 22), financial crisis and distress (n = 19), COVID-19 positive (n = 7), loneliness and missing family (n = 6), social boycott and pressured to be quarantined (n = 5), unable to return home due to lockdown (n = 2), COVID-19 related work stress (n = 3), alcohol unavailability (n = 2), alcohol addiction (n = 1), at quarantine center reasons not reported (n = 2), fear of being quarantined (n = 1), out of free will as reported in suicide note (n = 1), lockdown extension made the priest terribly depressed (n = 1), depressed due to postponement exams (n = 1), COVID-19 stress (n = 1),	NR
Rajkumar (2020)	Google search; NR	March 12 to April 11, 2020	23	Male (n = 20) and Female (n = 3)	NR	Fear of COVID-9 infection (n = 9), having influenza-like symptoms (n = 7), triggers unrelated to the disease (n = 6), alcohol dependence and withdrawal symptoms (n = 3), underwent stigmatization and ostracism despite testing negative (n = 1), Alleged work stress in a disaster management official (n = 1), separation from family due to transport restrictions (n = 1), Abrupt loss of a job in a migrant worker (n = 1), COVID-19 positive (n = 1), separation from family due to transport restrictions (n = 1), depression (n = 4)	Hanging (n = 10), Jumping from a height (n = 3), Cut-throat injuries (n = 3), Self- immolation (n = 1), and Medication overdose (n = 1)
Shoib et al. (2020)	Google search; NR	January 25 to April 18, 2020	34	Male (n = 28), Female (n = 4), missing (n = 2)	Young adult: 18 to 35 years (n = 18); Middle age: 36 to 55 years (n = 11); Older adult: > 56 years (n = 4)	Fear of COVID-19 infection (n = 16), misinterpretation of fever as COVID-19 (n = 9), depression and loneliness (n = 7), perceived stigma of COVID-19 infection (n = 4), financial problems and family dispute (n = 2), to save the village from infection (n = 1), anxiety after positive test results (n = 2), watching COVID-19 videos on social media (n = 2)	Hanging (n = 20), Burning (n = 4), Jumping from hospital, building and in front of train (n = 3), Slitting throat (n = 2), Drowning (n = 1), and Poisoning (n = 1)
Syed & Griffiths (2020)	Google search; NR	March 25 to May 17, 2020	27	Male	25 to 70 years	Alcohol withdrawal symptoms	Completed suicide: Poisoning (n = 8), Hanging (n = 6), Electrocution (n = 2), Cutting of wrists (n = 1), Drowning (n = 2), and Jumping from a building (n = 1) Attempted suicide: Jumping from a building (n = 1)
Manzar et al. (2021)	Purposive; adolescent and youth	February 15 to July 6, 2020	A total of 37 cases were reported in the study whereas 11 cases were from India	Male (n = 5) and Female (n = 6)		Pre-existing family stressors (n = 1), depression following quarantine (n = 1), lockdown restriction of movements (n = 3), unable to join or stressed by online schooling (n = 3), relationship complexities, *TikTok* addiction and depression following not getting likes (n = 1)	Hanging (n = 9), and Self-immolation (n = 1)
Kar et al. (2021)	Purposive; General people	Data were collected in two phases. Pre-lockdown: January 1^st^ to March 24, 2020 and Lockdown: March 25 June 30, 2020	A total of 769 cases were found; whereas 141 cases were from Bangladesh and 628 cases represent India	Male (n = 148/223) and Female (n = 75/223)	NR	Pre-lockdown: relational issues (n = 87/194), health issues (n = 32/194), financial stress (n = 31/194), and other issues (n = 44/194) During lockdown: relational issues (n = 196/373), health issues (n = 75/373), financial stress (n = 39/373), and other issues (n = 63/373)	Hanging (pre-lockdown vs lockdown: n = 135 vs 278), Poisoning (pre-lockdown vs lockdown: n = 36 vs 45), Fire arm (pre-lockdown vs lockdown: n = 25 vs 15) and others (pre-lockdown vs lockdown: n = 20 vs 53)
Panigrahi et al. (2021)	Google search and electronic search; NR	Data were collected in three phases. Pre-lockdown: February 1^st^ to March 24, 2020; Lockdown: March 25 to June 7, 2020 And Unlock: June 8, September 30, 2020	151	Male (n = 122) and Female (n = 29)	Mean age: 38.7(± 14.6) years	Quarantine/isolation (n = 74/143), fear of COVID-19 (n = 74/143), Stressed due to flu-like symptoms (n = 71/143), advised a COVID-19 test (n = 51/143), positive COVID-19 result (n = 41/143), financial loss (n = 28/143), stigma and discrimination (n = 24/143), unavailability of alcohol due to lockdown (n = 6/143)	Hanging (n = 72), Jumping from height (n = 29), Jumping in front of the train (n = 8), Poisoning (n = 8), Burning self (n = 5), Jumping into well (n = 3), Gun-shot (n = 3), and Slitting throat (n = 2)
Sripad et al. (2021)	Google search; COVID-19 infected individuals	June 30 to August 16, 2020	93	Male (n = 40), female (n = 15), and missing (n = 3)	Median age: 45 years (range 15 to 80)	Positive COVID-19 result (n = 58), Suspected with COVID-19 infection (n = 35)	Hanging (54%), Jumping from height (12.9%), Drowning (3.2%), Slitting throat/wrist (4.2%), and Poisoning (4.2%)
Pathare et al. (2020)	Google search; NR	Data were collected in two years. March 24 to May 3, 2019 and March 24 to May 3, 2020	369	During the pandemic: male (464)	Age group: < 30 (n = 102), 31 to 50 (n = 38), and > 50 years (n = 25)	Poor mental health (n = 18), poor physical health (14.1%), sexual harassment (2.5%), and fear of COVID-19, lockdown and/or person being quarantined (n = 157) These were statistically associated with suicidal behaviors in 2020 compared to 2019: Age between 31 and 50 years, male gender, married, and employed	Completed suicide: Hanging (2019 vs 2020: n = 76 vs 190), Poisoning (2019 vs 2020: n = 39 vs 25), Jumping in front of a train (2019 vs 2020: m = 17 vs 6), Drowning (2019 vs 2020: n = 10 vs 11), Jumping from height (2019 vs 2020: n = 20 vs 11), and Others such as Self-immolation, and self-infliction (2013 vs 2020: n = 19 vs 33) Attempted suicide: Hanging (2019 vs 2020: n = 0 vs 5), Jumping from height (2019 vs 2020: n = 3 vs 8), Poisoning (2019 vs 2020: n = 9 vs 8), Jumping in front of a train (2019 vs 2020: n = 3 vs 0), Drowning (2019 vs 2020: n = 1 vs 4), and Others such as self-immolation, and self-infliction (2019 vs 2020: n = 7 vs 11)
Ahmed et al. (2020)	Google search; NR	25 March to May 5, 2020	23	Male	28 to 70 years	Unavailability of alcohol and alcohol withdrawal symptoms	NR
Jahan et al. (2021)	Google search; Healthcare professional	NR	A total of 26 cases of healthcare professional globally were reported in the study where 8 cases represent India	Male (n = 3) and female (n = 5)	Age range 22 – 60 years	Work-related stress (n = 2), COVID-19 infection (n = 2), depression with/without suicidal tendencies (n = 2), mental disturbance (n = 1)	Hanging (n = 3), and Jumping from a building (n = 4)
Mamun et al. (2020)	Google search; celebrity	NR	The study reported Indian celebrity suicide during the COVID-19 and non-COVID-19 period whereas 7 cases were reported during the COVID-19 pandemic	Male (n = 3) and Female (n = 4)	NR	Depression (n = 5/7), bipolar disorder (n = 1/7), and Depression and loneliness (n = 1/7)	Hanging (n = 6), and jumping from building (n = 1)
Lathabhavan & Griffiths (2020)	Online news portal; Student	June 2, 2020	A 15 years old academic brilliance girl committed suicide. She was depressed due to not attending online classes or watching lessons on television. The TV was not functioning and she did not own a smartphone which made her think that her academic excellency would be greatly hampered	Female	15 years	Depression	Burning
Goyal et al. (2020)	Online news portal; NR	February 12, 2020	A 50 year-old man from Andhra Pradesh committed suicide due to fear of COVID-19 infection. He misunderstood his viral illness as COVID-19 and later on committed suicide by hanging on a tree	Male	50 years	Fear of COVID-19 infection	Hanging
Kar et al. (2020)	Online news portal; celebrity	NR	7	Male and Female	NR	Depression (n = 1), relationship complexities (n = 1), financial loss (n = 3), unemployment (n = 2)	NR
Hossain & Purohit (2020)	Online news	NR	A farmer lived in Uttar Pradesh committed suicide due to helplessness and worry by hanging on a tree. He lost his fertile land because of waterlogging along in a debt of 1.5 lacs. Besides, laborers refused to work in his land which triggered him to complete the suicide hierarchy	Male	52 years	Helplessness, worried	Hanging
Balaji & Patel (2021)	Google search	February 1, 2020 to May 31, 2020	370	Male (n = 250). females (n = 40), transgender (n = 1)	Age groups: 10 –19 (n = 15), 20–29 (n = 66), 30–39 (n = 71), 40–49 (n = 43), 50–59 (n = 31), above 59 (n = 21), and unspecified (n = 44)	Economic hardship (n = 108), fear (n = 63), isolation (n = 60), deprivation to return to loved ones or return home (n = 38), craving for alcohol (n = 28), domestic disputes (n = 17), aspirational disappointments (n = 14), discrimination and rejection (n = 12), restrictions to behaviors (n = 7), police violence and harassment (n = 6)	Cutting (n = 2), drowning (n = 14), drug overdose (n = 1), electrocution (n = 1), firearm (n = 1), jumping (n = 18), pesticide (n = 14), poisoning (n = 12), jumping in front of a train (n = 6), self-immolation (n = 11), unspecified (n = 27)
Khadse et al. (2022)	Google search; students	June 2020 to January 2021	42	Male (n = 18), female (n = 24)	Age groups: 11–13 (n = 6), 14–18 (n = 33), and 19–20 (n = 3)	Inability to access online education due to unavailability of smartphones or internet facilities (n = 19), inability to cope with online education (n = 15), scolded by parents for not paying enough attention in the online classroom and getting distracted by online games, videos, or social media sites (n = 5), and unknown reason (n = 3)	Hanging (n = 30), poisoning (n = 6), burning (n = 2), stabbing (n = 1), and unspecified (n = 3)

Note, some of the cases reported here included multiple suicide risk factors

**Table 2:** Quality assessment of the included articles

First author(s) and publication year	Documentation	Uniqueness	Educational value	Objectivity	Interpretation	Total
Dsouza et al. (2020)	1	1	1	1	1	5
Rajkumar (2020)	0	1	0	1	1	3
Shoib et al. (2020)	1	1	0	1	1	4
Syed & Griffiths (2020)	1	1	1	1	1	5
Manzar et al. (2021)	2	1	1	1	1	6
Kar et al. (2021)	1	1	1	1	2	6
Panigrahi et al. (2021)	1	1	0	1	2	5
Sripad et al. (2021)	1	1	0	1	1	4
Pathare et al. (2020)	1	1	0	1	2	5
Ahmed et al. (2020)	1	1	1	0	1	4
Jahan et al. (2021)	1	1	1	0	1	4
Mamun et al. (2020b)	1	1	0	0	1	3
Lathabhavan & Griffiths (2020)	2	1	1	1	2	7
Goyal et al. (2020)	0	1	1	0	1	3
Kar et al. (2020)	0	1	0	1	1	3
Hossain & Purohit (2020)	2	1	0	1	1	5
Balaji & Patel (2021)	1	1	0	1	1	4
Khadse et al. (2022)	1	1	0	1	1	4

### Suicide stressors

All studies reported suicide reasons or stressors. These were classed into four categories: (i) socio-demographic stressors, (ii) behavior and health-related stressors, (iii) COVID-19 related stressors, and (iv) psychopathological stressors.

#### Socio-demographic stressors

Only three studies recorded the socio-demographic factors as reasons for suicide [12, 21, 27]. A study that reviewed media reports from March 24 to May 3 2019 and March 24 to May 3 2020 reported 157 COVID-19-related suicides (128 completed suicides and 29 suicide attempts) aged 31 to 50 years. Of the 157 cases, 73 were male, 41 were married, and 86 were employed [12]. Unemployment appeared to be a key suicidal stressor in two (out of seven) cases of celebrity suicide [21]. Sudden unemployment in one migrant worker was found to be a stressor leading to suicide in a study of 23 cases [27].

#### Behavior and health-related stressors

##### Alcohol

A total of six studies reported alcohol-related suicide during the pandemic [15, 18, 19, 26, 27, 30]. Two of the studies included all of the suicide cases having alcohol-related issues [i.e. 27 cases reported by Syed and Griffiths [30] and 23 cases reported by Ahmed et al. [18]. Here all cases were due to alcohol withdrawal symptom-related stress. In the other four studies, 3/69 cases by Dsouza et al. [15], 5/144 cases by Panigrahi et al. [26], 28/370 cases by Balaji and Patel [19], and 3/23 cases by Rajkumar [27] reported the unavailability of alcohol and/or alcohol addiction as the suicide stressors.

##### Physical health

Physical health problems were reported to be a suicide stressor in two studies [12, 22]. A study comparing suicide cases in 2019 with 2020 found more physical health issues among individuals who died by suicide during the pandemic [12]. In addition, health issues were also recorded as stressors leading to suicide in 32/194 cases during the pre-lockdown period and 75/373 during the lockdown, in a press-media reporting study [22]. However, this latter study showed no significant difference between suicide pre-lockdown and during the lockdown.

#### COVID-19 pandemic related stressors

##### Fear of COVID-19 infection

Fear of COVID-19 was the most prominent stressor related to suicide, with a total of 343 cases reported in several studies [14, 15, 19, 20, 26–28]. India’s first suicide occurrence (reported above) was allegedly due to the fear of COVID-19 infection during the COVID-19 pandemic [14]. Another study of 69 cases reported fear of COVID-19 as the most prominent factor of suicide [15].

##### COVID-19 test results

COVID-19 test results also appeared to have influenced suicide. Several studies identified COVID-19 positive test results as a cause of suicide [15, 26–29] whereas Panigrahi et al. [26] reported that an individual being advised to take a COVID-19 test was a stressor that led to suicide [33.7% (51 out of 151)]. One individual who had negative COVID-19 results was reported to have died by suicide due to stigmatization and ostracism by the local community [27]. Two individuals were reported to have died by suicide due to anxiety following positive COVID-19 test results [28].

##### Quarantine/isolation

The COVID-19 quarantine/isolation period appeared to have a negative impact on individuals, which in turn can trigger suicide. Four studies reported that quarantine or isolation played a role in individuals’ suicides: being pressured or being afraid to be quarantined (6/69) and two died by suicide at quarantined centers (2/69) [15]; depression during quarantine (1/11) [25]; being quarantined or isolated (74/143) [26]; and among the 157 individuals reported by Pathare et al. [12] some died due to being quarantined (although the exact numbers were not reported).

##### Financial conditions

Financial conditions due to the COVID-19 lockdown greatly hampered nations’ economies all over the world. Of the included studies, six reported suicides by individuals who were financially distressed or experienced financial loss; 19/69 by Dsouza et al. [15], 2/34 by Shoib et al. [28], 31/194 by Kar et al. [22], 28/143 by Panigrahi et al. [26], 108/370 by Balaji and Patel [19], and 3/7 cerebrities by Kar et al. [21].

##### Miscellaneous

There were some miscellaneous stressors of COVID-19-related suicide in India such as: inability to willingly return home due to lockdown travel restrictions, [15, 19]; having influenza like symptoms [27]; undergoing stigmatization, discrimination, and ostracism [19, 26–28]; separation from family due to transport restrictions [27]; misinterpretation of fever as COVID-19 [28]; family disputes (Balali et al; [28]; saving the village from infection [28]; watching COVID-19 videos on social media [28]; online schooling [23, 25]; relationship complexities [21, 22, 25]; being suspected of having COVID-19 infection [29]; police violence and harassment [19]; and sexual harassment [12].

#### Psychopathological stressors

##### Depression and/or loneliness

A total of 8 studies indicated suffering from depression and/or loneliness as a trigger to suicide: 2/69 by Dsouza et al. [15], 4/23 by Rajkumar [27], 7/34 by Shoib et al. [28], 2/11 adolescents by Manzar et al. [25], 1/8 healthcare workers by Jahan et al. [20], 6/7 celebrities by Mamun et al. [7], and 1/7 celebrities by Kar et al. [21]. One study also reported depression as being the cause of suicide [24]. Depression was due to several factors such as lockdown, postponement of exams, quarantine, and *TikTok* addiction [15, 25]. However, one doctor was reported to have both depression and suicidal tendencies [20].

##### Stress

Three studies reported that work-related stress was a factor leading to suicide (3/69 by Dsouza et al. [15], 1/23 by Rajkumar [27], and 2/8 health workers by Jahan et al. [20]). Other studies found that some individuals were stressed due to flu-like symptoms committed suicide: 7/23 by Rajkumar [27] and 71/143 by Panigrahi et al. [26]. A total of three individuals died by suicide due to stress related to online schooling [25]. However, one case had had pre-existing family-related issues and one case had stress that led to death by suicide [25].

##### Miscellaneous

Other suicide-related stressors have been reported during the pandemic including poor mental health (one reported by Jahan et al. [20] and 18/369 by Pathare et al. [12]). One media case study also reported feelings of helplessness and worrying as causes of suicide [31].

### Methods of completing suicide

#### Hanging

Hanging was found to be the most used method of completing suicide in most studies [6–8, 12, 22, 23, 25–29]. More specifically: Kar = 278/391 (71.1%); Mamun = 12/16 (75%); Manzar = 19/37 (51.4%); Panigrahi = 72/151 (47.6%); Pathare = 190/295 (64.4%); Rajkumar = 10/18 (55.6%); Shoib = 20/32 (58.8%); Sripad = 50/93 (54%); Syed and Griffiths = 6/27 (22.2%; second most after poisoning); Khadse = 30/42 (71.42%); Lathabhavan et al. and Goyal et al. were single cases.

#### Jumping from something

A total of 90 victims jumped from something, comprising jumping from a high building (n = 70), jumping in front of a train (n = 17), and jumping into a well (n = 3) [7, 12, 20, 26–30].

#### Poisoning

Eight studies reported poisoning as a method of suicide during the COVID-19 pandemic [12, 19, 22, 23, 26, 28–30]. A total of 117 victims used this method and most commonly reported Kar et al. [22] who reported 45 cases among the general population during lockdown.

#### Drowning

Suicide by drowning was reported in five studies [12, 19, 28–30] [Pathare = 11/295 (3.7%); Shoib = 1/34 (2.94%); Sripad = 3/93 (3.2%); Syed and Griffiths = 3 /27 (11.1%); Balaji & Patel = 14/370 (3.78%)]. In one study, a total of 21 cases reportedly involved drowning although four survived and were considered as attempted suicides [12].

#### Burning and self- immolation

Burning oneself was reported to be a method of suicide in six studies [22–24, 26, 28]. A total of 57 individuals used this method of suicide during the pandemic. Self-immolation was reported in one study (n = 11/370) [19].

#### Miscellaneous

There were various other methods of completing suicide reported in the literature including medication overdose [(n = 1) [19]; (n = 1) [27]], electrocution [(n = 2) [30]; (n = 1) [19]], firearm (n = 1) [19], pesticide (n = 14) [19], stabbing (n = 1) [23], and gun-shot (n = 3) [26]. Also reported during the COVID-19 pandemic included individuals dying by suicide by cutting or slitting throat in a few studies [19, 26–29] [Panigrahi: n = 2; Rajkumar: n = 3; Shoib: n = 2; Sripad = 4/93 (4.2%); Balaji & Patel: n = 2]. One individual slit their wrist in their suicide [30].

### Methods of attempting suicide

Two studies reported the methods used in attempted suicide [12, 30]. These methods included hanging [5 out of 36 (13.8%)], poisoning [8 out of 36 (22.2%)], drowning [4 out of 36 (11.1%)], self-immolation [11 out of 36 (30.5%)], jumping from height [8 out of 36 (22.2%)], and jumping in front of a train [no number reported] [12]. Jumping from a building was reported in one case [30].

## Discussion

The present systematic review was conducted to explore the stressors that lead to suicide and the methods used to die by suicide among the Indian population during the COVID-19 pandemic. Several studies have been conducted that have examined pandemic suicide in India, but to understand the overall scenario, a more systematic approach was needed.

In India, males were found to die by suicide more than females during the pandemic. Previous studies have reported a similar finding. For instance, a global English newspaper review of 18 cases between January 1 to April 30, 2020 (15 suicides, two suicide attempts, and one homicide-suicide) reported 17 were males [32]. Similarly, media report studies from Bangladesh and Uganda have reported a higher number of males committing suicide compared to females [33–36]. Males appear to be more likely to die by suicide in the current pandemic. Traditional social factors such as the need to appear masculine may be an obstacle to sharing their feelings and seeking support, increasing the risk of suicide. Therefore, there is a need for emotional and mental health support for men to improve their mental health to reduce the suicide rate.

In addition, unemployment status and abrupt job loss were also found to be suicide stressors. Similarly, in Bangladesh a study reported lockdown-related financial recession as the most prominent factor of suicide during the early period of the pandemic [33]. Another study reported that poverty was one of the contributing factors for individuals dying by suicide [35]. In addition, a longitudinal study covering 63 countries explored the relationship between suicide and unemployment, estimated a 20%–30% increase in unemployment-associated suicide rate over a 12-year period (2000–2011) [37]. Given this unprecedented situation, some preventive measures such as financial safety nets including food, housing, loans, and additional focus on active labour market programs, might effectively reduce suicide-related to financial conditions [38].

Unavailability of alcohol and/or withdrawal of alcohol symptoms and poor physical health were significantly reported as stressors leading to suicide. Alcohol consumption in India has increased over the past few years [39]. More specifically, approximately 5.4 billion litres of alcohol were consumed in India in 2016, whereas it was 6.5 billion litres in 2020 [39]. This means that Indian individuals are drinking alcohol more frequently than in the past. Therefore, not being able to access alcohol because of countrywide lockdown-related factors appears to have triggered suicide in extreme cases. One study in India reported 27 cases of suicide, in which 26 cases completed the suicide due to the fact they could not cope with alcohol withdrawal symptoms [30]. To prevent this kind of unnecessary death, the government (as well as non-governmental organizations) may attempt to adopt preventive strategies such as monitoring alcohol intake, spreading safe drinking messages, providing access to safe drinking, and disseminating crisis resource messages organizations [40]. However, individuals with physical medical conditions are at a greater risk of suicide than those who are not. Similar findings have also been reported in previous studies. For instance, a census-based follow-up study of over one million individuals found that individuals with a lot of physical medical conditions were at a three-fold greater risk of suicide than those who had no limitations in their physical activity [41]. Another study among the US general public found that multiple physical conditions such as traumatic brain injury, sleep disorders, and HIV/AIDS significantly exacerbated suicide risk [42].

The first case of COVID-19 suicide in India reported fear of COVID-19 as a significant factor [9]. Similarly, in a neighbouring country (i.e. Bangladesh), fear was also reported as a stressor of the first case of COVID-19-related suicide [43]. This finding suggests that fear is the most reported factor for dying by suicide among individuals. A press-reporting media study comprising 69 Indian reports concluded that fear of COVID-19 was the most prominent risk factor for suicide [15]. Individuals who suspect they have COVID-19 and those advised to test for COVID-19 have also been reported as reasons for suicide. For instance, one study indicated that half of the Indian suicide cases emerged within one week of COVID-19 diagnosis confirmation, and half of the suicides occurred at COVID-19 centres [29]. Therefore, counselling, raising awareness, and campaigning should be given the highest priority to combat such suicides.

Quarantining and isolating are considered primary level measures to combat COVID-19. A previous study showed its effectiveness during the COVID-19 pandemic. For example, non-quarantining communities showed an incidence and death rate of 96% and 76%, whereas after adhering to quarantine measures, the rate diminished to 44% and 31% [44]. However, studies have also shown the negative impact of mass quarantine or isolation among individuals, reporting it as a risk factor for suicide [33, 35]. However, a study among US adults found no associations between physical distancing and increased suicidal behaviors during the pandemic [45]. It is anticipated that suicide incidence might be (in part) due to social disconnection. Therefore, telecommunication and social connection might reduce the rate of suicide in this context. Other stressors related to COVID-19 include family disputes, watching COVID-19 videos on social media, online schooling, and relationship complexities or issues. Online schooling-related suicide has also been reported in other countries. A mother-son suicide pact was reported as an online learning issue during the pandemic [6].

It is well-established that psychological suffering (i.e., depression, anxiety, stress, loneliness, insomnia, etc.) can lead to suicide in extreme cases. Moreover, approximately 90% of individuals who die by suicide have a mental health disorder. The present findings also supported this. The present study reported depression, loneliness, stress, *TikTok* addiction, and poor mental health as suicide stressors that intensified suicidal ideation. A recent systematic review found suicidal ideation prevalence rate ranging from 5% to 19%, where depression, anxiety, stress, insomnia, suicidal thoughts history, suicide attempt history, and family history of committing suicide were the major attributors to suicidal behaviour [10]. Therefore, mental health symptoms may play a significant role in suicide, and intervention to relieve mental health symptoms are vital during the pandemic to reduce suicide.

Although hanging was the most preferred method of suicide, individuals also died by suicide by poisoning, jumping off or into something, burning, slitting throat or cutting wrists, drowning, medical overdose, and gunshot. A 5-year study from one of the largest cities of India recruiting over 5000 cases reported different method of suicide among the victims. They found poisoning as the most common method of suicide among males followed by hanging, firearms, burns, drowning, and jumping from a height, whereas for females, it was also poisoning followed by hanging, burns, drowning, jumping from a height, and firearms [46].

## Limitations

The present study has a number of limitations. First, the data comprised only cases from media reports, and some of the suicide reports might have been used in multiple studies which means the numbers of cases may not be as accurate as they could be and may lead to overestimated figures concerning suicide stressors or methods of suicide. Second, with often limited information provided by the media case report, sophisticated analysis is almost impossible. Third, information reported by media in the included studies may be biased and/or simplistic because none of the reported studies performed psychological autopsies. In addition, the suicide data extraction from the media reports may be biased or subjective due to the suicide expertise of the authors involved in the respective studies. Finally, due to some media stories adhering to the reporting guidelines for suicide as provided by the World Health Organization may also have led to omission of some important information (details about stressors or methods, for example) that provide additional insight into suicide during the pandemic.

## Conclusions

Suicide is of serious concern during the current pandemic situation, and reducing it is a significant challenge for a country such as India, where more than 380 individuals have died daily due to suicide during the pandemic. Findings from the study demonstrated the method and alleged risk factors of suicide in a developing country during the COVID-19 pandemic. Findings from this research suggest multiple reasons for suicide during the pandemic and knowledge of such factors could aid in developing suicide prevention strategies focusing the most vulnerable cohorts inside and outside India. Further study is suggested to conduct observational studies using a quantitative approach to explain the risk factors of suicide rather than a qualitative approach.

## Data Availability

All data used in generating the manuscript is presented within the manuscript.
